# Omentectomy in Addition to Bariatric Surgery—a 5-Year Follow-up

**DOI:** 10.1007/s11695-017-2576-y

**Published:** 2017-02-02

**Authors:** Daniel P Andersson, Daniel Eriksson-Hogling, Jesper Bäckdahl, Anders Thorell, Patrik Löfgren, Mikael Rydén, Peter Arner, Johan Hoffstedt

**Affiliations:** 1 Department of Medicine, Karolinska University Hospital Huddinge, Karolinska Institutet, Stockholm, Sweden; 2 Department of Surgery, Karolinska Institutet, Ersta Hospital, Stockholm, Sweden

**Keywords:** Visceral fat, Gastric bypass, Randomized, Clinical trial, Insulin resistance

## Abstract

**Aim:**

Omentectomy in addition to bariatric surgery has been suggested to improve metabolic outcome but short-term (6–24 months) studies have refuted this notion. We investigated whether there was any long-term impact of omentectomy.

**Methods:**

Forty-nine obese women underwent gastric bypass surgery and were randomly assigned to omentectomy (*n* = 26) or not (*n* = 23). They were re-examined after 5 years including dual-energy X-ray absorptiometry for body composition, blood pressure and blood sampling.

**Results:**

There were no significant differences between the two groups at baseline (*p* = 0.07–0.93) or 5 years post-operatively (*p* = 0.15–0.93) regarding weight, BMI, body composition, HOMA-IR, plasma cholesterol, HDL cholesterol, or triglycerides.

**Conclusion:**

In agreement with previous shorter studies, removal of the greater omentum in addition to GBP is not associated with metabolic benefits after long-term follow-up.

## Introduction

Abdominal obesity, which reflects the amount of visceral adipose tissue, has been identified as an independent risk factor for type 2 diabetes and cardiovascular disease [1]. The increased metabolic risk of excess visceral adipose tissue is probably due to multiple underlying mechanisms. Björntorp described in 1990 that the portal drainage of visceral adipose tissue to the liver results in increased levels of metabolites in visceral obesity that may have profound effects on hepatic metabolism [2]. In particular, non-esterified fatty acids have been proposed to be of importance as visceral adipose tissue displays increased lipolysis compared to subcutaneous depots [3]. This prompted the hypothesis that removal of visceral adipose tissue in conjunction with bariatric surgery could have additional positive metabolic effects. The major omentum, which constitutes a substantial portion of the visceral adipose tissue compartment, is easily accessible during surgery. In 2002, our group published a pilot study designed as a randomized controlled trial in 50 subjects undergoing adjustable gastric banding with or without simultaneous removal of the omentum. After 2 years, we found a significantly improved glucose tolerance and insulin sensitivity in the group undergoing omentectomy [4]. However, since then, several randomized controlled trials with up to 2 years follow-up have reported no effects of either omentectomy in addition to bariatric surgery or omentectomy alone, as discussed previously [5]. The purpose of the present study was to investigate, in a previously described cohort [5], whether positive metabolic effects of omentectomy together with gastric bypass could be observed over a longer follow-up of 5 years.

## Material and Methods

The inclusion and exclusion criteria, patient characteristics before, and 2 years post-operatively have previously been described in detail [5]. Briefly, 81 obese women scheduled for open gastric bypass were randomized to operation with or without removal of the greater omentum. For pre- and post-surgical investigations, the subjects reported to the research unit in the morning after an overnight fast. Height, weight, waist circumference, and blood pressure were measured. Body composition was measured with dual-energy X-ray absorptiometry (DEXA) using a GE-Lunar iDXA (GE Healthcare, Madison, WI, USA). The software (enCORE version 14.10.022) was used to determine body fat content in different locations and also to estimate visceral adipose tissue (EVAT) and subcutaneous adipose tissue (ESAT) depots in the android region, as previously described and discussed [6, 7]. A blood sample was collected for subsequent analysis of glucose, insulin (Insulin, ELISA, Mercodia Uppsala, Sweden), cholesterol, high-density lipoprotein (HDL), and triglycerides. Insulin resistance was estimated by the homeostasis model assessment of insulin resistance (HOMA-IR). Subjects were contacted by phone every 6 months during the first 2 years post-operatively and were asked to report their current weight. The study was approved by the regional ethical review board. All participants gave their written informed consent. The statistical software JMP version 12.1.0 (SAS Institute Inc. Cary, NC. US) was used to compare groups with unpaired *t* test in per-protocol analysis. The primary endpoint 2 years post-operatively in the randomized controlled trial was insulin sensitivity measured by hyperinsulinemic euglycemic clamp and this parameter was used for power calculation to define the number of subjects needed to detect a clinically significant difference between the groups [5]. In this follow-up study, we had insulin sensitivity measured by HOMA-IR as primary endpoint and did not perform any power calculation.

## Results

**Fig. 1:**
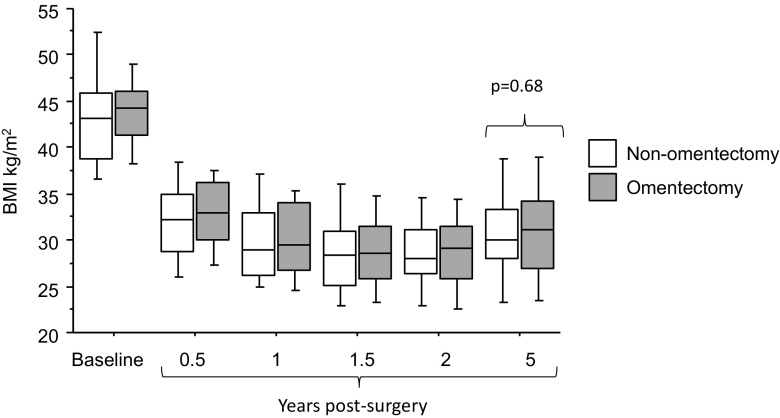
BMI development following bariatric surgery with or without omentectomy. Values before surgery and during the first 2 years post-operatively have been published previously [5].

Out of the 81 subjects initially included in the study, 26 omentectomized and 23 non-omentectomized completed the follow-up examinations 5 years post-operatively. The was no statistical difference between the subjects that were examined after 5 years and the subjects that were lost to follow-up regarding baseline age, weight, BMI, glucose or insulin. As expected, there was a significant improvement in all anthropometric and metabolic parameters at 5 years after compared to before surgery in both groups (Table 1). The average weight of the removed omentum from the 26 omentectomized subjects was 525 ± 246 g with a removed omentum weight/baseline body weight ratio of 0.0045 ± 0.0023. There was a significant weight regain between the 2- and 5-year follow-up examinations (omentectomy 79 ± 15 kg to 85 ± 19 kg *p* = 0.02 and non-omentectomy 79 ± 15 kg to 85 ± 16 kg *p* = 0.0002, but no significant difference in body weight change between the two groups (*p* = 0.15). Figure 1 shows that there were no significant changes in BMI between the groups over the study period. In addition, there were no significant differences between the omentectomy and non-omentectomy group regarding total body fat, EVAT, ESAT, fasting plasma glucose, −insulin, HOMA-IR, plasma lipids, or systolic blood pressure (Table 1. *p* = 0.10–0.98). A small but statistically significant higher diastolic blood pressure was observed in the omentectomy compared to the non-omentectomy group (see Table 1, *p* = 0.03).

**Table 1:** Clinical characteristics. Values are mean ± SD. Analyzed with unpaired *t* test. Baseline characteristics have previously been published [1, 5]

	Baseline			5-year follow-up		
	Non-omentectomy	Omentectomy	*P* value	Non-omentectomy	Omentectomy	*P* value
*N*	41	40		23	26	
Age (years)	41 ± 9	43 ± 9	0.42	48 ± 10	49 ± 9	0.72
Weight (kg)	119 ± 16	121 ± 15	0.52	85 ± 16	85 ± 19	0.94
BMI (kg/m^2^)	43.1 ± 5.4	44.3 ± 4.7	0.27	30.7 ± 5.2	31.4 ± 6.0	0.67
Waist to hip ratio	1.00 ± 0.07	1.00 ± 0.06	0.64	0.93 ± 0.07	0.92 ± 0.08	0.85
Total body fat %	53 ± 4	54 ± 3	0.41	43 ± 7	43 ± 8	0.93
Estimated visceral adipose tissue (kg)	2.39 ± 0.87	2.35 ± 0.78	0.84	0.90 ± 0.55	0.91 ± 0.66	0.98
Estimated subcutaneous adipose tissue (kg)	3.93 ± 1.00	3.89 ± 1.39	0.90	2.07 ± 1.07	2.35 ± 1.34	0.41
Systolic blood pressure (mmHg)	133 ± 17	137 ± 17	0.23	125 ± 14	134 ± 22	0.09
Diastolic blood pressure (mmHg)	84 ± 9	85 ± 8	0.67	76 ± 9	82 ± 10	0.03
P-glucose (mmol/L)	5.5 ± 1.1	5.6 ± 1.3	0.77	5.0 ± 0.7	5.0 ± 0.6	0.82
P-insulin (mU/l)	14.7 ± 7.5	18.2 ± 9.4	0.07	5.2 ± 2.4	6.2 ± 2.8	0.15
P-cholesterol (mmol/l)	4.8 ± 0.9	4.9 ± 0.9	0.62	4.2 ± 0.9	4.3 ± 0.8	0.73
P-HDL cholesterol (mmol/l)	1.1 ± 0.2	1.2 ± 0.3	0.55	1.6 ± 0.4	1.6 ± 0.5	0.57
P-triglycerides (mmol/l)	1.5 ± 0.7	1.5 ± 0.7	0.93	1.0 ± 0.6	0.9 ± 0.3	0.65
P-NEFA (mmol/l)	0.75 ± 0.24	0.81 ± 0.17	0.22	0.6 ± 0.3	0.7 ± 0.3	0.52
P-apolipoprotein B (g/l)	0.91 ± 0.23	0.97 ± 0.25	0.28	0.82 ± 0.22	0.83 ± 0.22	0.83
P-apolipoprotein A1 (g/l)	1.22 ± 0.20	1.23 ± 0.22	0.85	1.56 ± 0.33	1.55 ± 0.37	0.89
P-alanine aminotransferase (U/L)	34.2 ± 17.6	34.6 ± 16.5	0.91	19.7 ± 7.2	18.2 ± 6.8	0.46
P-aspartate aminotransferase (U/L)	28.9 ± 12.6	31.0 ± 11.9	0.45	22.7 ± 4.5	23.1 ± 8.1	0.81

## Discussion

This study shows that there are no long-term beneficial effects of omentectomy in conjunction with gastric bypass operation 5 years post-operatively. To our knowledge, this constitutes the longest follow-up time that has been reported using this type of intervention [5].

The rationale for why a long-term follow-up of these subjects was necessary is that following a rapid initial weight loss after gastric bypass, there is often a significant weight regain already, 1 year after the intervention [8]. In addition, even though a majority of subjects experience remission of type 2 diabetes initially, a relapse in diabetes can sometimes be seen over time and it is possible that increased amounts of visceral adipose tissue could play a role in this [8]. However, our present data, demonstrating that omentectomy does not provide long-term benefits, suggest that diabetes relapse may depend on other mechanisms.

The reason for why removal of a large proportion of the visceral adipose tissue does not lead to positive metabolic effects have been discussed previously [5]. Most importantly, the potential positive effects of removing approximately 0.5 kg of adipose tissue might be concealed by the overall much larger adipose tissue reduction following gastric bypass operation or the average small proportion that it constitutes of the baseline body weight (0.45%). Interestingly, 5 years post-operatively, there was no significant difference in EVAT between the omentectomy group and non-omentectomy group. This can also be an explanation why there is no difference in metabolic parameters between the groups. Admittedly, a limitation regarding this observation is that our measurement of visceral adipose tissue is an estimation based on calculations made from DEXA examinations, and although this estimation is a valid measure of visceral adipose tissue [9], it has to our knowledge never been validated in subjects that have undergone omentectomy. An additional weakness is that the present study population size was based on a follow-up from a previous study and not based on a power calculation. However, no statistically beneficial changes were found with omentectomy, and we consider that, even if a larger study might find statistically significant changes between the groups, there would probably not be any clinically significant beneficial changes in the omentectomy group.

In conclusion, omentectomy in conjunction with gastric bypass operation does not confer any positive effects on weight, insulin sensitivity, blood lipids, or blood pressure over a long period of time. With the present study and previous studies in mind, the relevance of omentectomy has been definitely addressed.
